# Use and Isolation of Urinary Exosomes as Biomarkers for Diabetic Nephropathy

**DOI:** 10.3389/fendo.2014.00149

**Published:** 2014-09-26

**Authors:** Luca Musante, Dorota Ewa Tataruch, Harry Holthofer

**Affiliations:** ^1^Centre for BioAnalytical Sciences (CBAS), Dublin City University, Dublin, Ireland

**Keywords:** exosomes, extracellular vesicles, urine, diabetic nephropathy, podocyte

## Abstract

Diabetes represents a major threat to public health and the number of patients is increasing alarmingly in the global scale. Particularly, the diabetic kidney disease (nephropathy, DN) together with its cardiovascular complications cause immense human suffering, highly increased risk of premature deaths, and lead to huge societal costs. DN is first detected when protein appears in urine (microalbuminuria). As in other persisting proteinuric diseases (like vasculitis) it heralds irreversible damage of kidney functions up to non-functional (end-stage) kidney and ultimately calls for kidney replacement therapy (dialysis or kidney transplantation). While remarkable progress has been made in understanding the genetic and molecular factors associating with chronic kidney diseases, breakthroughs are still missing to provide comprehensive understanding of events and mechanisms associated. Non-invasive diagnostic tools for early diagnostics of kidney damage are badly needed. Exosomes – small vesicular structures present in urine are released by all cell types along kidney structures to present with distinct surface assembly. Furthermore, exosomes carry a load of special proteins and nucleic acids. This “cargo” faithfully reflects the physiological state of their respective cells of origin and appears to serve as a new pathway for downstream signaling to target cells. Accordingly, exosome vesicles are emerging as a valuable source for disease stage-specific information and as fingerprints of disease progression. Unfortunately, technical issues of exosome isolation are challenging and, thus, their full potential remains untapped. Here, we review the molecular basis of exosome secretion as well as their use to reveal events along the nephron. In addition to novel molecular information, the new methods provide the needed accurate, personalized, non-invasive, and inexpensive future diagnostics.

## Background

Diabetes, chronic kidney disease (CKD), and cardiovascular disease (CVD), [DCC] are significant causes of morbidity and excess mortality and with an alarmingly increasing global trend. Together, they constitute the DCC-disease complex that accounts for major human suffering and already more than 15% of the economic burden on all Healthcare systems (1, 2) Consequently, there are ever increasing academic, industrial, and societal investment needs in combating this worsening epidemic.

The DCC complex shares key symptoms (e.g., microalbuminuria, high cholesterol, and elevated blood pressure) and risk factors (e.g., genetic traits, exercise, and dietary deficiencies) (3, 4). Many lines of evidence show that an early common denominator for DCC diseases is malfunction of the kidney (glomerular) filtration barrier and, particularly, its epithelial component, the podocyte. Recent milestone discoveries have revealed molecules, mechanisms, and pathways of the podocyte, which associate directly with in the onset and progression of the disease (5, 6).

The alarming statistics of DCC highlights the importance of understanding the molecular and cellular pathways thereof. Important advances in understanding the mechanisms leading to the loss of kidney function have been made. Accordingly, abnormal glucose level is known to distort several molecular pathways like the renin–angiotensin system (RAS), inflammatory cytokines (transforming growth factor β (TGF-β) and tumor necrosis factor-α (TNF-α)), protein kinase C (PKC), and the Janus kinase pathway and, oxidative stress mediators, such as nicotinamide adenine dinucleotide phosphate oxidase (NADPH oxidase). All of these have been associated with pathogenesis of diabetic nephropathy (DN) [for dedicate review see Ref. (7–9)] while, the clinical management of patients to protect the kidney function in diabetes are not satisfactory (10, 11). Therefore, more accurate early diagnostics and better disease management is needed.

A key consideration relates to the availability of clinically relevant biomarkers for personalized disease management. Notably, the value of albumin excretion rate to urine, presently an important indication of CKD, is decreased by the following observations. First, diabetic patients presenting with microalbuminuria already show advanced damage in kidney filtration barrier associated with disease progression (10, 11). Second, one-third of diabetic patients developed diabetic kidney damage (nephropathy; DN) soon after the onset of microalbuminuria, and this was not dependent on the presence of relevant proteinuria (12). Finally, the albumin assay methodology in use has shown serious flaws for the presence of fragmented and biochemically modified albumin (12). Furthermore, up to 10% of general public shows occasional albumin leakage into urine.

## Extracellular Vesicles: Biogenesis, Classification, and Functions

**Figure 1:**
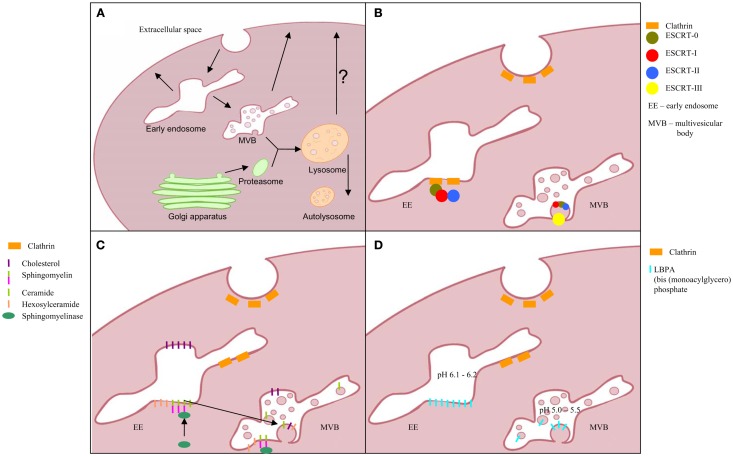
**Exosomes biogenesis; (A) multivesicular body formation; intraluminal vesicles formation pathway based on: (B) ESCRT complex involvement, (C) transformation of sphingomyelin into ceramide, and (D) triggered by phospholipid lysobisphosphatidic acid (LBPA) in acidic pH**.

Extracellular vesicles (see Figure 1) are spherical structures composed with a lipid bilayer carrying a cargo of proteins and nucleic acids, released by cells into extracellular space. This term comprises different classes of particles, which had been assigned by van der Pol and colleagues into three major groups: exosomes, microvesicles (MV), and apoptotic bodies (13). This classification subjects all membrane derived vesicles (shed vesicles, exosome-like vesicles, membrane particles, ectosomes, and retrovirus vesicles) into one group and endosomal pathway derived exosomes into other group. The distinction is not perfect as viruses can hijack Multivesicular Body (MVB) pathway and occur also inside of exosomes (14). Apoptotic blebs constitute a separate class as their presence is not associated with a continuously living cell, but with programed cell death process. Although more comprehensive classification based e.g., on size, density, sedimentation force, or vesicle-group specific biomarkers exists, these properties also overlap between EVs populations (15).

*Biogenesis of exosomes* includes inward budding of vesicles into endosomal lumen to become MVBs fusing with the cell membrane and simultaneously releasing exosomes into extracellular space (Figure 1) (16). Unlike the vesicles shedding directly from cell membrane, exosomes contain distinct cargo, closely mirroring the inner compartments of their cells of origin.

Frequently presented exosome diameter ranges from 40 to 100 nm and up to 150 nm (13, 15, 17). Unfortunately, this wide range includes a variety of other EV classes and cannot be considered as a solid identifier. The well-characterized pathway of exosome biogenesis includes proteins of the ESCRT complex (the endosomal sorting complex required for transport), which is also shared by lysosomal pathway of degradation. Ubiquitinated proteins are recruited by proteins of this complex and clustered in intraluminal vesicles (ILV, future exosomes) within an endosome to yield MVB (16).

Proteins associated with this pathway are grouped into four main complexes (ESCRT-0, -I, -II, and -III) and a class of accessory components (ALIX). ESCRT-0 is built by STAM1 and Hrs, which recognize the clathrin presence on the surface of early endosome as well as the ubiquitin on degradation-directed proteins. Hrs interact with Tumor susceptibility gene 101 (TSG101), an ESCRT-I protein and thus hires this complex. Together with ESCRT-II, ESCRT-I is responsible for membrane deformation into buds, whereas, ESCRT-III recruited by ALIX is required for scission of the bud neck. For more detailed description, the recent comprehensive reviews of ESCRT machinery and role of each protein (16, 18, 19) should be consulted.

This pathway clearly describes the mechanism of directing and anchoring protein cargo into ILV as well as the vesicle formation. Interestingly, considering importance of ESCRT machinery in exosome biogenesis, only 7 from 23 of ESCRT genes after silencing in HeLa-CIITA-OVA cells presented significant influence on this pathway. Furthermore, knock-down of four of these (ESCRT-III complex proteins CHMP4C, VPS4B, VTA1, and ALIX) induced increase in exosome release (20). Thus, alternative pathways of ILV formation have been proposed. Trajkovic and colleagues described a ceramide-dependent process of ILV inward budding in mouse oligodendroglial cell line (Oli-neu cells) (21). In these cells, ceramide is taken from microdomains of early endosomal membranes by the action of sphingomyelinases and lead to fusion of microdomains into larger domains favoring a domain-induced budding. However, the study was focused on membrane trafficking of the proteolipid protein (PLP) and thus do not describe the mechanism of cargo entrapping in ILVs. This pathway seems to yield the vesicles for extracellular release as the PLP is found in exosomes. Alternative pathway suggests a role for lysobisphosphatidic acid (LBPA) in ILV formation. However, lack of LBPA in released vesicles may indicate the determination of lysosomal degradation pathway or different mechanism of ILV formation in which LBPA is involved indirectly (18, 22). Another ESCRT-independent pathway is suggested by Theos et al. (23). In their publication, a ubiquitin-independent mechanism of directing cargo into ILV was postulated using the melanosomal protein Pmel17 as a thus contrasting the proposed ceramide or LBPA pathway.

In the literature, there is disagreement over the next steps after ILV formation within the MVBs. Accordingly, fusion with lysosome/proteasome before directing the MVB to the plasma membrane has been proposed. This could explain the presence of a variety of lysosome specific proteins (proteasome subunits, cathepsin E and B, kallikrein 1, DPPIV, lipoprotein lipase, prolyl 4-hydroxylase, transmembrane protease, serine carboxypeptidase I, and lysosomal-associated membrane protein 1 and 2) within EVs. On the other hand, urinary extracellular vesicle (UEV) protein databases contain a number of molecules of apoptotic blebs (e.g., histones) and MV in general (e.g., cholesterol and diacylglycerol) (24, 25). This suggests that any EV populations may in fact be highly heterogeneous. This raises a real need to better define exosomes as those in diameter <100 nm recovered in pellet 200,000 g.

Term “*microvesicles*” includes all structures budding directly from the cell membrane by rearrangement of the lipid bilayer, especially in phospholipid distribution and with help of cytoskeletal proteins (26). Full mechanism of vesicles budding from plasma membrane is not well known. Nonetheless, direct involvement of some ESCRT machinery proteins like TSG101 into process of vesicle shedding not related with hijack performed by viruses has been presented. Those results are coming from mammalian cell cultures (HeLa, HEK293T, and A549) and from *C. elegans* embryos, suggesting more wide-spread mechanism (22, 27). This once again raises the question whether TSG101 is suitable as exosome-specific marker.

*Apoptotic bodies* are released into extracellular space as the consequence of apoptosis – the programed cell death. Their diameter range varies from 20–500 nm to 1,000–5,000 nm (15, 28). In this biological process, activated by p53 and caspases, the apoptotic cell is splitting into multiple apoptotic blebs, which are autolyzed by release of lysosomal content or phagocytozed without inflammatory reaction (29). DNA degradation in the nucleus is characteristic for this process and, thus, histones associated with nucleic acid can be used as biomarkers for apoptotic blebs. However, a great number of histone fragments have also been found in exosomes (24, 25).

International Society for extracellular vesicles (EVs) emphasized in 2012 that most EV preparations are heterogeneous and also contain membrane budding vesicles and apoptotic bodies. Thus, an urge for standardized protocol has been expressed (30).

**Figure 2:**
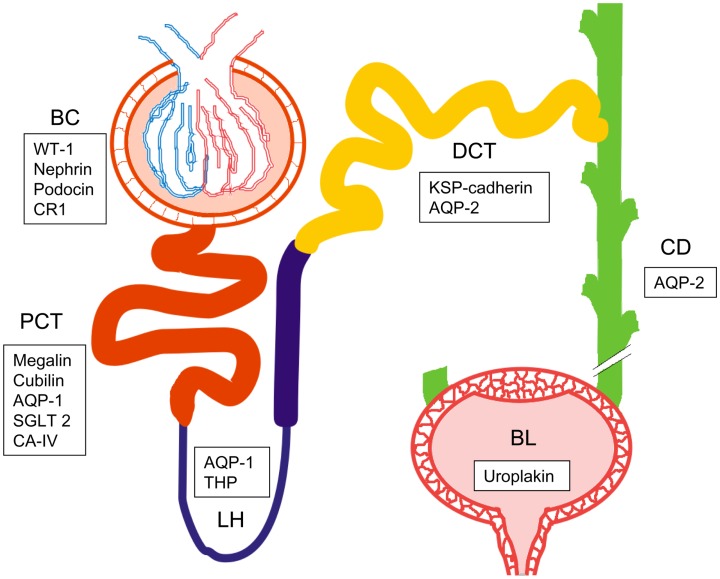
**Schematic picture of the urogenital route with one nephron and bladder**. Bowman’s capsule (BC), proximal convoluted tubule (PCT), loop of Henle (LH), distal convoluted tubule (DCT), collecting duct (CD), and bladder (BL). List of specific biomarkers for each nephron segment are presented in frames. WT-1, Wilm’s tumor 1; CR1, complement receptor 1; AQP1, aquaporin 1; SGLT2, sodium-glucose linked transporter 2; CA-IV, carbonic anhydrase IV; THP, Tamm-Horsfall protein; KSP-cadherin, kidney specific cadherin; AQP2, aquaporin 2.

Importance of exosomes increased after discover of their role in cell-to-cell communication. Although it is a function reserved for exosomes, there are also publications describing membrane derived vesicles as a special type of messengers (15) Thus, vesicles released by one cell, can be taken up by other distal cell (31) and the respective contents recognized as useful and important message (32). Of special note is that microRNAs of vesicle cargo can directly affect processes in the target cell by silencing specific genes. Moreover, vesicles can transport not only to neighboring cells but also on relatively long distance (33). It is intriguing to speculate that in the special case of kidney nephrons, substantial distant communication between the epithelial cells can take place (see Figure 2). This distant signaling is used by healthy donor-cells as well as cancer cells, making it an efficient benefit for cancer progression and tumor expansion (32). On the other hand, promising data are presented on using vesicles as treatment of human cells *in vitro*. Silencing RNAs (siRNAs) were thus introduced into recovered exosomes, and exposed to HeLa and HT1080 cells *in vitro*, efficiently silencing targeted genes (34).

Thus, being EVs carrier of information a relative long list of functions has been attributed to them depending on the cell (tissue) type specificity. Characterization of exosomes secreted from B-lymphocytes and dendritic cells showed that these vesicles may carry the full set of proteins to induce an immunoresponse (35–37). Moreover, EVs isolated from various body fluids and stored blood can induce the production *in vitro* of inflammatory cytokines like interleukin (IL)-1, tumor necrosis factor α (TNF-α), IL-23a for example (38, 39). In addition to exosomes, also MV have been proposed to serve as mediators of inflammation (40). Interestingly, MV concentration increases in diseases involving vascular and microvascular organ injury like diabetes (41–43). In addition to inflammation, MVs may regulate e.g., the endothelial production of reactive oxygen species as mediated by NADPH oxidase (44, 45). Conversely, exosomes released by cells under oxidative stress can induce neighboring cells to have a higher resistance to ROS, therefore becoming more tolerant to the damage of oxidative stress (46). Interestingly, very recently Gildea and colleagues (47) showed that in their *in vitro* model of exosomes, cell communication between proximal and distal tubules, proximal exosomes could induce distal and proximal duct cells to limit ROS production after stimulation with a dopamine receptor agonist. Thus, not only this report reinforces the concept of cell-to-cell communication and consequently modulator of signal pathway but also extends it to a new level: nephron segment-to-segment communication.

## Urinary Vesicles as Potential Source for Biomarkers

One of the first publications describing vesicles in the urine dates back to 1987 when Wiggins et al. described the presence of lipid MV with procoagulant activity (48) in urine of rabbits in nephrotoxic nephritis. This was followed by series of reports by Scherberich (49) to describe shedding of membrane – bound enzymes in a vacuolar membrane morphology (50–500 nm in diameter) from the proximal tubule in patients with kidney injury after administration of potentially nephrotoxic drugs. Later on Pascual et al. characterized vesicles carrying complement receptor 1 (CR1) in urine unequivocally shown to originate from the podocyte (50). Consequently, these were proposed as markers of podocyte injury. However, the interest and follow-up remained low until the first comprehensive description of exosomes in healthy urine by Pisitkun et al. (51). This publication can be considered the milestone in the UEV field, opening possibilities for accurate cell type-specific molecular changes in nephrology and urology.

The unprecedented characterization of the vesicle associated proteins and protein fractions (apparently due to enzymatic degradation within urine) using especially advanced mass spectrometry certainly laid a solid basis for their potential applications in clinical diagnostics and disease follow-up and, consequently, brought UEVs to a wider public awareness. Since then the number of publications exploring urinary exosomes and/or other MV have rapidly and exponentially increased. Furthermore, in addition to focus strictly on exosomes, other types of vesicles have been described in urine including, among others, exosome-like vesicles (52) and shedding vesicles (53). Notably, all the UEV subsets have their distinct intracellular secretory pathways. These are also reflected in the more or less carefully defined isolation protocols, respectively, while their distinct potential for diagnostics-disease management largely overlaps. This emphasizes the need to establish robust protocols to enable UEVs isolation and analysis *in toto* for such purposes.

Proteomic profiling of UEVs has identified a surprising number, abundance, and distinct character of proteins originated from cells all along the nephron epithelium: from the visceral glomerular epithelial cell (podocyte) to the proximal tubule, thick ascending limb of Henle, distal convoluted tubule, and the collecting duct (54–58). Additionally, mass spectrometry analysis has described amino acid sequences originating of proteins of the lower part of the genitourinary path including the urinary bladder and prostate gland (51, 52, 54–58).

Consequently, analysis of urinary exosomes has proven to be an attractive, non-invasive approach to the physiological state of epithelial cells of distinct origin. This clearly offers a true opportunity for future “fluid biopsy” to complement – or up to replacing – information from the invasive kidney biopsies or cystoscopy.

Accordingly, Prunotto et al. (59) were recently able to pinpoint biomarkers and follow the physio-pathological state of specific cell types by enriching and characterizing vesicles released specifically from the podocyte. Interestingly, their almost 1,200 protein identifications discovered 14 previously unidentified urinary proteins. Many of these were subsequently classified (by gene ontology analysis) as brain-specific proteins whose expression in the kidney was then confirmed. Unless these specific proteins are from vesicles gaining direct passage through the kidney glomerular filter, this evidence highlights, once again, similarities between podocyte and set of neuron specific proteins (60). Thus, it seems obvious that exosomes (together with the other vesicular structures found in the urine) will genuinely offer an interesting new approach to dig into the distinct nephron segment-specific molecules and detect changes induced by disease processes (61).

In addition to proteins, UEVs carry a wide range of genetic information molecules like mRNA – encoding proteins native to all nephron regions and, especially small RNA (62, 63) species. While the few targeted methods may show promise of better RNA yields (64–66), in general methods for RNA isolation also need serious optimization. Interestingly, the lipid-bilayered UEVs appear to be protected of the ubiquitous RNases, suggesting that UEVs do have a true physiological role in downstream signaling.

While the finding of the variety of viable RNA species in UEVs is intriguing, the full meaning of this needs to be better understood. It is interesting to speculate again a role for special downstream modulation as a form for distant cell-to-cell communication and, hence, for biomarkers and targets for treatment.

Recently, CD2AP (of the podocyte) specific mRNA has been proposed as biomarker of kidney disease (67). Among the 194 microRNA species thus far reported from exosomes, 45 had appeared to present with significant association to blood pressure modulation (68). Similarly, microRNA-29c in UEVs correlates with renal function and degree of general fibrosis thus emerging as a potential urinary marker of renal fibrosis. Barutta and colleagues recently showed that the expression of miR-130, miR-145, miR-155, and miR-424 were significantly alterated in type 1 diabetic patients with incipient DN (69). This result deserves full validation and additional studies. Finally, in addition to RNA and miRNa, UEVs may serve as carriers of mitochondrial DNA, which has shown to be significantly decreased in DN patients (70) to suggest that an altered bioenergy supply of kidney cells may promote ROS production and localized inflammation both contributing to progress of renal damage [reviewed in Ref. (71)].

For DN, considerable advances have been achieved with biomarkers derived from UEVs. Accordingly, Wilm’s tumor-1 (WT-1) protein expression appears to increase with the decline of the renal function in DN (72). Interestingly, urinary WT-1, most likely derived from the podocyte, may thus qualify as a simple marker of podocyte injury rather then a specific marker of DN (73–75).

The amount and activity of dipeptidyl peptidase IV (DPPIV/CD26) in urinary MV coming from proximal tubule cells was shown to positively correlate with progression of DN in type 2 diabetic patients (76) suggesting an early tubular impairment, which may be considered an early marker of renal damage even before the onset of albuminuria.

Zubiri and colleagues (77) carried out a proteomic quantitative analysis on urine samples from DN patients in advanced disease stages (CKD stages III–V). A panel of three potential proteins were included: protein fragment of alpha-1-microglobulin/bikunin precursor (AMBP), isoform 1 of histone-lysine N-methyltransferase (MLL3), and voltage-dependent anion-selective channel protein 1 (VDAC1). These showed differential expression in the samples studied. If verified in larger sample sets, this represents still another promising finding of UEVs as biomarkers.

Finally, very recently in the animal models of DN an increase of MVs secreted from podocytes before the onset of albuminuria (78) was reported. This finding highlights the potential of MV and other UEVs as early markers of glomerular injury.

Furthermore, changes in WT-1 were seen in DN (72), focal segmental glomerulosclerosis, and in minimal change nephropathy (73, 74). Studies performed in other kidney diseases like IgA nephropathy and thin basement membrane nephropathy (TBMN) (79) have identified four candidate proteins (aminopeptidase N, vasorin, ceruloplasmin, and α1 anti-trypsin) associated to UEVs, which appear differently expressed in disease. Among the four protein candidates ceruloplasmin showed highest sensitivity and specificity to distinguish IgA from TBMN. However, for full verification more samples are needed.

Several candidate biomarkers proposed have not followed yet. For example, Fetuin-A has been proposed as predictive biomarkers for acute kidney injury (80) while no further evidence to validate the original finding has been presented. Similarly, aquaporin-1 (AQP-1) was presented as a novel specific urinary biomarker to follow-up ischemia reperfusion injury (81) while further studies are needed for validation.

## Methods for Extracellular Vesicle and Urinary Extracellular Vesicle Isolation

Although the number of publications describing a variety of protein and RNA based potential disease biomarkers is still increasing, there is no consensus of standard protocols for EV isolation.

In most studies of UEVs, the method of differential centrifugation is used for their isolation with the aim to obtain enriched exosomal fraction. However, minor technical variability is often described, which may lead to significant impact on final results (82). Notably, there is no agreement in cell and cell debris removal from either cell culture media or from biofluids. Some studies use centrifugations with low different relative centrifugal forces (RCF) (lower than 10,000 *g*) whereas, others prefer direct approach with force over 16,000 *g* for cell debris and apoptotic bleb removal (29, 37). Unfortunately, little systematic analytics of the discarded fractions have been published.

Still another point of inconsistency is the ultracentrifugation step in which forces achieved may vary from 100,000 *g* up to 200,000 *g*. In this respect, it is important to note that Thery and colleagues could show that sedimentation point for most types of vesicles may range between 100,000 and 200,000 *g* and highlighted that obtained fractions are heterogeneous (37).

Relative centrifugal force (*g*) given in most publications is describing only force applied on the bottom of vial. Lack of technical information like type of rotor (fixed angle or swing bucket), diameter, volume, and viscosity of the sample do not allow estimated the force affecting upper part of media/biofluid. This leads to variable yield obtained from similar type of samples, which may cause challenges in downstream applications (83) of the material isolated.

In some studies, ultrafiltration is applied *between* differential centrifugation steps. Here also lack of standardization leads to use special filter device with varying porous filter from 0.1 or 0.2 μm up to 0.8 μm (26, 29, 30) and the appropriate moment for use (after low force centrifugation step or directly before ultracentrifugation). Additionally, little systematics has been devoted to lost yield caused by clogs of the filter membrane or variability of exosome size (82). In terms of membrane-derived vesicles, removal at this step is questionable as size of these structures vary from 20 up to 1,000 nm (30, 37), and we do not know if particular disease can have impact on their size distribution.

Lately, commercial products for exosome enrichment have been released (ExoQuick^®^System Biosciences and Total exosome isolation reagent ^®^Invitrogen), which allow to omit the ultracentrifugation step (84). It is worth to emphasize that regardless the product names, they are enriching heterogeneous populations of vesicles but their use carry also disadvantages. They are cost-efficient only for very concentrated samples like plasma and, furthermore, these methods are poorly applicable for diluted biofluids (e.g., urine). Additionally, they may cause co-precipitation of most abundant soluble proteins in media/biofluid and thus are not suitable for protein profiling using Mass Spectrometry.

Methods for isolation of UEV are mainly based on differential centrifugation with some modification due to the intrinsic and peculiar characteristics of urine as sample. The first subfraction was reported by Pisitkun et al. (51) while detailed analysis of their efficiency, specificity for the targeted fractions has not been systematically studied. Moreover, efficiency of these methods so as not to lose valuable UEV fractions remains to be defined. Furthermore, as stated above, the true practical importance of obtaining highly pure subpopulations still remains elusive. A major reason for this is that while the intracellular pathways in the formation of various vesicle classes have been well established (20), their contents indiscriminately reflect events interior of the cell. Thus, from the applications point of view to reflect cell type-specific changes, the whole variety of UEVs may be more important to harvest for analysis. As an example, CR1, among the full array of complement pathway-specific proteins has been recognized among the 1,195 protein identifications with the method used (pullout by podocyte-specific antibody) (59). Presently available methods, furthermore, do not distinguish abundance of each of the protein species among the full UEV repertoire leaving opportunity for method improvement. Likewise, the same problem of cross-contamination with UEV subspecies, loss of substantial fractions of specific target vesicles usually occurs when the separation is made on physical characteristics of the vesicles e.g., by their density buoyancy (52, 79, 85–87).

The most abundant proteins in the UEVs include e.g., human serum albumin (or, more precisely, its fragments) and Tamm–Horsfall Protein (THP) (produced in cells of ascending limb of the loop of Henle), Aquaporin-1 (of the proximal tubulus), Aquaporin-2 (distal tubulus and collecting duct), uroplakin (urinary bladder) in addition to apolipoproteins, immunoglobulins, and other soluble proteins (54, 88, 89). Their presence in the whole UEV proteome has been well established and thus, already provide source for nephron segment-specific detailing.

Tamm–Horsfall glycoprotein represents a special case among UEV proteins as it actively forms urinary protein meshworks to entrap a variety of vesicles (89). This may reflect a physiological role for THP to modulate UEV abundance. Consequently, adherence to THP may result in huge differences in the UEV subtype yields if the THP “contaminant” is not removed from urine at the early steps of UEV isolation. It is interesting to note that our recent observations clearly show high fluctuations of daily, intraindividual THP levels in the urine (unpublished data). As THP is secreted mainly from the distal parts of nephron, this could reflect the proposed physiological role of THP to modulate UEV access to more downstream targets. Interestingly, UEVs are known to display a distinct surface proteome (as opposed to their respective “cargo”) consisting of specific proteins also capable for receptor binding. Thus, modulating the UEV availability may be constantly in control by other secreted urinary proteins like THP.

The conventional isolation protocol of UEVs starts with a pre-concentration step, followed by series of differential centrifugations or direct ultrafiltration (mostly to reduce the final volume). For density gradients to distinguish UEV subclasses, the same density layer proteins “characteristic” of exosomes (i.e., with marker protein TSG101), MV (marker prominin-1), apoptotic markers (marker histone proteins) along with matrix proteins (collagens, fibronectin fractions), soluble proteins (THP/HSA), or apolipoproteins (E, D, A1, AIV) can be found indiscriminately. Up to now no study has evaluated the possibility of vesicle–matrix, vesicle–soluble protein, vesicle–vesicle, and vesicle–lipoprotein interaction or whether this is a technical artifact caused by the pre-enrichment step. For comprehensive evaluation of UEV usefulness for physiological analytics and not to cross-contaminate them in different density gradient layers, systematics to yield optimized protocols should be achieved. Moreover, no studies have carefully addressed the localization of UEV protein contents themselves: are they present on outer surface or inside the vesicles is important to understand their possible role in distant signaling along the nephron. Our recent results indicate considerable differences in these two UEV domains to suggest of distinct “address tag” and/or effector functions, respectively. Notably, one report has shown that in urine of patients with light chain amyloidosis, multiple myeloma, and monoclonal gammopathy of undetermined significance (MGUS), light chains were found on the exosome surfaces (90).

## Bottlenecks of UEV Isolation and Future Aspects

As reported previously, one of the factors limiting wide implementation of UEVs includes the ambiguity of the isolation methods in use. The current golden standard method initially established by Pisitkun et al. (51) includes differential centrifugations followed by treatment of the yield either with a reducing agent (like dithiothreitol) followed by a second ultracentrifugation step (9, 51, 54, 55, 61, 63, 77, 91) or/and by centrifugation in density cushion/gradient (52, 88, 92, 93) or/and by size exclusion chromatography (94) or/and by ultrafiltration devices (9, 62, 64) or/and ExoQuick precipitation reagent (7, 54). All these protocols have their advantages and limitations (Table 1) while aim to obtain pure population of vesicles, especially exosomes. At the same time these protocols attempt to eliminate the undesirable interference from THP also known as uromodulin (UMOD). THP forms polymers in urine that sediment during centrifugation and easily entraps vesicles (95). Our results fully recapitulate this problem and call for better solutions to avoid it (manuscript in submission). The most commonly used and fast approach uses a reduction step by dithiothreitol (DTT) to reduce the 24 disulfide bonds of THP in order to increase the yield of vesicles after ultracentrifugation. Unfortunately, this extra step further complicates the UEV isolation process and, despite the de-folding of THP, this still co-sediments with UEVs. In fact such a denaturation step can cause aggregation due to the exposure of hydrophobic domains in an aqueous environment. Furthermore, this leads to alterations in the three-dimensional structure of THP to minimize its free energy by optimized hiding of hydropobic groups and, conversely, exposing as many polar residues as possible in the unnatural, non-urine environment. The hydrophobic groups can also interact with each other to form intermolecular hydrophobic bonds further catalyzing aggregation and co-precipitation with abundant UEVs attached. This mechanism is plausible and was recently exploited to deplete plasma from high abundant disulfide-rich proteins for mass spectrometry analysis (96).

**Table 1:** **Overview of the main methods to isolate urinary vesicles**.

Reference	Short method description	Advantages	Disadvantages	
Barutta et al. (69)	Differential centrifugation	Vesicles enrichment as a pellet	No fixed conditions for: 1. number of centrifugations; 2. relative centrifugation force; 3. time; 4. rotor type; 5. sample volume; 6. temperature during centrifugation; and 7. presence/absence of protease inhibitors	Not applicable for large volume of samples and not suitable for large cohort of patients. Expensive equipment like ultracentrifuge and HPLC are necessary to implement UEVs enrichment
Gildea et al. (47)	Differential centrifugation	
Kalani et al. (72)	Differential centrifugation	
Lv et al. (65)	Differential centrifugation	
Musante et al. (98)	Differential centrifugation + CHAPS treatment	Protein activity prevention	Labor intensive	
Gonzales et al. (54)	Differential centrifugation + DTT treatment	Increase of the exosomal yield in the ultracentrifugation pellet from THP interference	Not suitable for protein activity assessment designated samples. Incomplete removal of THP from the ultracentrifugation pellet	
Fernandez-Llama et al. (95)	Differential centrifugation + DTT treatment	
Wang et al. (55)	Differential centrifugation + DTT treatment	
Cheng et al. (63)	Differential centrifugation + DTT treatment	
Rood et al. (91)	Differential centrifugation + SEC	Vesicle separation according to size	Labor intensive	
Rood et al. (91)	Nano-membrane filtration	Concentration and removal of soluble proteins	Differences in removal of bigger (>0.22 μm or more) vesicles without assessment of their importance for biomarkers screening. Adsorption of vesicles on the surface of ultrafiltration membrane	
Merchant et al. (89)	Differential centrifugation + microfiltration	
Miranda et al. (62)	Differential centrifugation + nanofiltration	
Principe et al. (57)	Differential centrifugation + ultrafiltration	
Prunotto et al. (59)	Differential centrifugation + ultrafiltration	
Hogan et al. (52)	Differential centrifugation + sucrose gradient	Vesicle separation according to density	Extremely labor intensive	
Raimondo et al. (116)	Differential centrifugation + sucrose gradient	
Ramirez-Alvarado et al. (90)	Ultrafiltration + differential centrifugation	Vesicle separation according to density	Extremely labor intensive	
Zubiri et al. (77)	Exoquick	No need of ultracentrifugation step. Suitable for extraction of RNA and DNA	Precipitation solution is expensive especially when applied for large volumes of urine and number of samples. For proteomic analysis, extra steps are necessary to remove the interference from precipitating agent	
Alvarez (64)	Exoquick	
Musante et al. (unpublished)	Hydrostatic dialysis	Inexpensive, large volume of urine		

Factors in UEV isolation conditions such as variable pH, temperature, and ionic strength can further influence the ratio of the interaction between hydrophobic residues themselves and/or interaction with the solvent (97). These evidences may directly explain the differences in results obtained and call for better standardized isolation protocols. Our hydrostatic dialysis (HD) method (manuscript in submission) utilizes simple dialysis with defined cut-off membrane directly for the whole void urine and achieves a superior yield of UEVs while simultaneously eliminating THP contamination and, furthermore, standardizes the electrolyte content of the sample. By additionally eliminating the need for ultracentrifugation steps and yielding reasonable volumes e.g., for biobanking purposes at low cost, a very high throughput of samples can be obtained easily.

We have also replaced DTT treatment with a more gentle zwitterionic detergent, 3-[(3-cholamidopropyl)dimethylammonio]-1-propanesulfonic (CHAPS) to better preserve the tertiary structure stabilized by disulfide bonds (98). CHAPS is a non-denaturating detergent designed to disrupt non-specific protein interactions and preserving protein conformation (99). Moreover, the average size of CHAPS derived micelles is ~6 kDa and, therefore, it can easily be removed by a simple dialysis step. With the introduction of CHAPS we also manage to preserve the activity of two indicator enzymes associated to UEVs-dipeptidyl dipeptidase IV (DPPIV) and nephrilysin (NEP) (99). Especially, NEP activity benefits of the replacement of DTT to CHAPS. As an obvious explanation for these results, the extracellular domain of NEP contains 12 cysteine residues, forming 6 disulfide bridges. Four of these are located within the catalytic domain while the other two participate in maintaining the structure consisting of two multiply connected folding domains embracing a central cavity containing the active site (100). Evaluation the proteolytic activity of NEP after reduction and re-oxidation of disulfide bridges during dialysis highlights that an incorrect re-folding of the enzyme could have happened leading to an impairment of NEP activity. Therefore, for the aforementioned reasons, CHAPS can be considered an optimized substitute of DTT if remaining physiological activity in the sample is of importance.

However, both UEV treatments, with DTT and CHAPS, did not completely remove the THP interference from the pellet recovered. It is, however, to remember that THP is a glycosylphosphatidylinositol (GPI) anchor protein synthetized exclusively by the epithelial cells at the ascending limb of Henle’s loop. It is transported to the apical side of the plasma membrane and released in the tubular lumen by a still undefined protease action [see Rampoldi et al. for more details, (101)].

As a GPI-anchored protein and in such abundance in the urine, THP may be involved in the formation of membrane cargo vesicles and, perhaps, also in the signal transduction by providing a receptor structure for ligands like cytokines (102–105). In support of its involvement as receptor and vesicle trafficking, THP was recently found in basolateral translocation process followed by secretion in the circulation. This was, interestingly, associated with recovery of kidney function in a murine model of ischemic acute kidney injury (106). Thus it should not come as a surprise if THP, as a GPI-anchored protein, is a key constituent of MV shed in the tubular lumen by direct budding of plasma membrane or as secreted through the endosomal pathway. More detailed studies to elucidate these proposed functions are warranted.

Although differential centrifugation is widely used to recover UEVs it has several shortcomings. One major drawback is the less-optimal efficiency of recovery in the distinct pellet fraction (107) but also in the final supernatant. Rotor type, g-forces applied, and centrifugation time significantly affect the final yield (108). Moreover, such a methodology does not allow high throughput of samples.

Alternative methods proposed to avoid these limitations are the use of customized nano-membrane concentrators and microfiltration disk membranes (57, 59, 62, 76, 77, 89, 91, 109, 110). Exosomes and abundant soluble protein fractions bind to the membrane surfaces of these device non-selectively and irrespective of the chemical composition of the filters. This leads to excessive obstruction of the nano-membrane. Thus, only smallish volumes of urine can be processed and, therefore, centrifugation still remains the preferential method to enrich and isolate UEVs. However, independently from the method used some common problems remain. First, the volume of urine to be processed is limited while the time required is excessive.

We propose that most direct benefit emerging from understanding of EV biology will come from individualized measurements: personalized datasets of efficiency, side-effects, and general performance of medications. Furthermore, it is easy to speculate that simple measurements based on non-invasive sampling of biofluids like urine will prove to be very important in disease progression, prognostics, and disease management in general.

**Figure 3:**
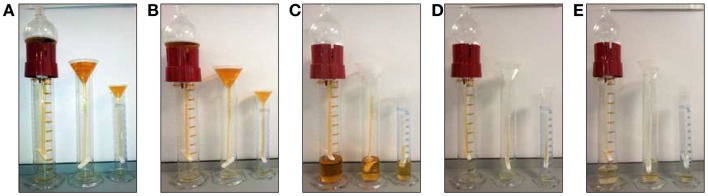
**Hydrostatic dialysis systems set up for large (0.5–1 l) medium (200–600 ml) and small volume (10–100 ml) of urine**. The dialysis membrane tube (MWCO 1,000 kDa) is connected to the separating funnel and/or a normal funnel. The bottom end is sealed with a universal dialysis tube closure. The separating funnel is filled in with supernatant from 2,000 *g* centrifugation **(A)**. The hydrostatic pressure of urine in funnel pushes the solvent (water) trough the meshwork of dialysis membrane and liquid below the MWCO, which fells to the bottom of the cylinder **(B)**. When urine is concentrated up to 7–8 ml **(C)** the funnel is re-filled with milliQ water (~200 ml equivalent to 25 volumes of concentrated urine), which flushes away all analytes left inside the tube **(D)**. This dialysis step is kept going until the solution is completely clear from yellow pigments (as internal natural control) and concentrated to a desired final volume **(E)**. The whole process has been named hydrostatic dialysis. Finally, it is possible to observe the diffusion of urine from the tube and precipitation at the bottom of the cylinder **(E)**.

Among the main limitation of working on UEV we would like to emphasize the dilution of the sample, limited amount of starting material (biobanking) and the extreme range of reference interval for analytes (urine is a waste product) responsible of a massive intra- and inter-individual analytical variability (111) In order to overcome such limitations, we recently designed and characterized an alternative approach for vesicle enrichment using basic, cost-efficient laboratory equipment we named our system HD from its operating principle (Figure 3).

In HD, urine samples are initially centrifuged at a RCF of 2,000 *g* to remove cells, bacteria, cellular casts, and the bulk of the prevalent THP macropolymers. Thereafter, the resulting supernatant is poured in the dialysis system consisting of a funnel connected with simple dialysis membrane with molecular weight cut-off (MWCO) of 1,000 kDa. The cut-off mol weight is easy to modify if needed for special purposes.

The hydrostatic pressure created by the sheer column of urinary solution is strong enough to push the solvent through the mesh of dialysis membrane together with all constituents, often containing unnecessary contaminants below the selected MWCO. This step simultaneously concentrates the sample, while the funnel is re-filled with deionized water and/or buffer of choice to rinse away all the analytes below the selected MWCO. This step optimizes and standardizes both sample concentration, electrolyte content, and sample volume to be suitable directly e.g., to biobanking of urine even from large volumes of starting material.

Full characterization of this methods revealed that it can enrich UEVs with far superior efficiency and minimal loss of vesicles on the dialysis membrane and in a short time compared to serial centrifugations.

We established that the filtration rate in HD is proportional to the volume of urine contained in the funnel with an average of 75 ml per hour of urine filtrate which takes also into consideration the fact that for large volume of urine (>200 ml) the system may loose some of its efficiency due the adsorption of soluble protein and/or vesicles in the dialysis. We found, however, that for the volume of 200 ml of urine, up to 18% of TSG101 and 45% of THP signal, respectively is adsorbed in the dialysis membrane. However, this method is a gentle way to concentrate first and then dialyze samples. In fact, urinary concentration already takes place at 1 g gravity at atmospheric pressure of sea level with respect to the 3,000 *g* RCF needed for the nano-membrane concentrator with 60 psi of special atmosphere of nitrogen pressure (64, 89, 91).

As urine is by far the simplest biofluid to collect in large quantities for biobanking purposes, our method vastly simplifies the process and practical volumes can easily be achieved for storaging.

However, it is worth to notice that urine in normal condition is a diluted solution and membrane protein most likely associated to UEVs represents an estimated 3% of the urinary proteome (112). Thus, by using HD the void urine can be collected and the whole volume processes soon after the first centrifugation. We have thus far handled up to 500 ml of urine within 24 h without changing in the protein pattern obtained. Moreover, several HD device can be run simultaneously and with minimal costs.

Notably, this new approach also allows normalizing the physical and chemical parameters of the retained solutions by eliminating all the urinary constituents’ analytes below the chosen MWCO. As recently reported, different sample compositions have an effect on the viscosity of the samples, which distorts the recovery of vesicles as obtained by differential centrifugation (113). Although urine is a diluted solution with a density and viscosity close to water we noticed that when the tip of the dialysis bag is immersed in water the stream of urine flowing from the tube sank on the bottom of the cylinder. Factors affecting the viscosity of urine include temperature, protein content (114), and hydration status (115). These are of pivotal importance when a comparative analysis between different subjects and patients’ samples with different grade of hydration and proteinuria are compared and a differential centrifugation protocol is employed.

In conclusion, although more than a decade has passed, interest and number of publications have been soaring, the simple UEV isolation still meets several challenges to overcome. Urine collection and methodology to enrich vesicles need to be optimized especially for clinical settings of sample collection and storaging. One particular challenge to overcome includes definition of suitable housekeeping genes and proteins to yield comparable results. Large scale validation of biomarkers derived from UEVs similarly remains to be accomplished. However, it is apparent that comprehensive understanding of the role of UEVs, both their surface proteome and internal cargo molecules and respective roles in distant addressing and cell-to-cell communication even to distant downstream location has started while careful future optimization of protocols and methods as well as standardization are needed.

## Conflict of Interest Statement

The authors declare that the research was conducted in the absence of any commercial or financial relationships that could be construed as a potential conflict of interest.

## Abbreviations

Alix, ALG-2 interacting protein; AMBP, alpha-1-microglobulin/bikunin precursor; CHAPS, 3-[(3-cholamidopropyl)dimethylammonio]-1-propanesulfonic; CHMP4C, charged multivesicular body protein 4C; CKD, chronic kidney disease; CR1, complement receptor 1; CVD, cardiovascular diseases; DCC, diabetes chronic complications; DN, diabetic nephropathy; DNA, deoxyribonucleic acid; DPPIV, dipeptidyl peptidase IV; DTT, dithiothreitol; ESCRT, endosomal sorting complex required for transport; EV, extracellular vesicles; GPI, glycosylphosphatidylinositol; HD, hydrostatic dialysis; HRS, hepatocyte growth factor-regulated tyrosine kinase substrate; HSA, human serum albumin; ILV, intraluminal vesicles; LBPA – lysobisphosphatidic acid; miRNA, micro ribonucleic acids; MLL3, histone-lysine N-methyltransferase; MVB, multivesicular body; MWCO, molecular weight cutoff; NEP, nephrilysin; Oli-neu cells, oligodendroglial cell line; PLP, proteolipid protein; Pmel17, melanosomal protein; RCF, g – relative centrifugal force; SEC, size exclusion chromatography; siRNA, silencing ribonucleic acids; STAM1, signal transducing adapter molecule 1; THP, Tamm–Horsfall protein; TSG101, tumor susceptibility gene 101; UMOD, uromodulin; VDAC1, voltage-dependent anion-selective channel protein 1; VPS4B, vacuolar protein sorting 4 homolog B; VTA1, vacuolar protein sorting-associated protein; WT-1, Wilm’s tumor-1 protein.
